# Integrated Clinical-Molecular Risk Stratification in Diffuse Large B-Cell Lymphoma: Machine Learning Survival Analysis

**DOI:** 10.2196/84636

**Published:** 2026-08-21

**Authors:** Jin Zhao, Xiaolian Wen, Li Ma, Liping Su

**Affiliations:** 1 Department of Hematology Shanxi Province Cancer Hospital/ Shanxi Hospital Affiliated to Cancer Hospital, Chinese Academy of Medical Sciences /Cancer Hospital Affiliated to Shanxi Medical University Taiyuan, Shanxi Province China; 2 Shanxi Provincial Key Laboratory of Lymphoma Precision Diagnosis and Treatment Research Taiyuan, Shanxi Province China

**Keywords:** diffuse large B-cell lymphoma, prognostic model, machine learning, transcriptomics, overall survival, relapse

## Abstract

**Background:**

The clinical outcomes of diffuse large B-cell lymphoma (DLBCL) are highly heterogeneous. While clinical indices like the international prognostic index (IPI) are widely used, their predictive accuracy remains limited. The integration of molecular features with clinical characteristics holds promise for developing more precise prognostic models to improve risk stratification and personalize treatment strategies.

**Objective:**

This study aimed to systematically identify key factors influencing overall survival (OS) and relapse in patients with DLBCL by leveraging publicly available transcriptomic data and clinical information. The goal was to construct and validate a high-precision risk-prediction model by using machine learning methods to aid in individualized clinical decision-making.

**Methods:**

We curated clinical and transcriptomic data from the GSE31312 cohort. A baseline clinical model was first constructed using multivariate Cox regression. Key genes associated with prognosis were identified through univariate Cox and survival analyses. Subsequently, 3 machine learning survival models, namely, fast survival support vector machine (FastSurvivalSVM), gradient boosting survival analysis (GBSurvival), and random survival forest (RSF), were trained and evaluated using 5-fold cross-validation. The interpretability of the optimal model was further elucidated using Shapley Additive Explanations (SHAP) methodology.

**Results:**

The baseline clinical model confirmed age, elevated lactate dehydrogenase, Eastern Cooperative Oncology Group score, Ann Arbor stage, and B symptoms as independent risk factors for OS and relapse-free survival, with a C-index of 0.65-0.67. At the molecular level, genes such as *PSMG4* and *CRY1* were significantly associated with poor OS, while *TMEM182* and *SPIRE1* were prominent in relapse prediction. Among the machine learning models, FastSurvivalSVM demonstrated the best overall performance, achieving an area under the curve of 0.791 for 1-year OS prediction and 0.774 for 1-year relapse prediction. SHAP analysis revealed that both clinical (eg, IPI and age) and molecular (eg, *PSMG4* and *SPIRE1*) features were critical drivers of the model’s predictions.

**Conclusions:**

This study successfully developed a multidimensional risk prediction model that integrates clinical and molecular characteristics for DLBCL. The FastSurvivalSVM model showed superior performance in predicting mortality and relapse risks. The interpretability analysis uncovered key prognostic factors, providing a valuable tool for personalized risk management and new theoretical insights for future mechanistic research.

## Introduction

Diffuse large B-cell lymphoma (DLBCL) is one of the most prevalent subtypes of non-Hodgkin lymphoma, accounting for more than 30% to 40% of cases globally [1,2]. With the widespread adoption of immunochemotherapy regimens such as rituximab, cyclophosphamide, hydroxydaunorubicin (doxorubicin), oncovin (vincristine), and prednisone or prednisolone, the overall survival (OS) rate of patients has significantly improved compared to the past. However, approximately one-third of patients experience relapse or refractory disease, leading to an extremely poor prognosis [3,4]. The median survival time for these patients is often less than a year, posing a major challenge in clinical treatment [5,6]. Despite recent advancements in novel targeted therapies and immunotherapies, accurately identifying which patients will experience relapse or mortality remains highly challenging. Achieving effective risk stratification at the initial diagnosis to predict individualized risks of death and recurrence, thereby providing a reliable basis for selecting clinical treatment strategies and monitoring therapeutic efficacy, has become a core scientific issue in the field of DLBCL.

Traditional clinical prognostic tools have played a significant role in the risk assessment of DLBCL [7]. The international prognostic index (IPI), introduced in the 1990s, has been widely used in clinical practice. It incorporates factors such as age, clinical stage, Eastern Cooperative Oncology Group (ECOG) performance status, lactate dehydrogenase (LDH) levels, and the number of extranodal sites to stratify patient prognosis [8,9]. However, with advancements in treatment modalities, the predictive accuracy of IPI has gradually declined, making it challenging to effectively differentiate between high-risk and low-risk populations in certain patient groups [10]. Additionally, while immunophenotype-based molecular markers (such as *CD10*, B-cell lymphoma 6 [*BCL6*], Multiple Myeloma Oncogene 1 [*MUM1*], and Forkhead box protein P1 [*FOXP1*]) and molecular subtyping (Germinal Center B-cell-like/Activated B-cell-like subtypes) hold some value in clinical applications, individual markers often have limited predictive power and fail to capture the complex biological heterogeneity of DLBCL [11]. These limitations result in some high-risk patients not being promptly identified at initial diagnosis and pose a risk of overtreatment among low-risk patients.

With the rapid advancement of multiomics and high-throughput sequencing technologies, numerous studies have unveiled significant differences at the transcriptomic, gene mutation, and signaling pathway levels in patients with DLBCL [12,13]. Evidence indicates that aberrant expression of specific genes is closely associated with patient survival. For instance, molecules involved in cell-cycle regulation, metabolic reprogramming, and immune evasion may serve as critical drivers affecting the risk of relapse and mortality [14,15]. Compared to traditional clinical indicators, molecular characteristics can reflect tumor biological behavior at a more refined level, offering new perspectives for risk prediction [16-18]. However, the predictive power of individual genes is limited, and results across different studies often lack consistency. This inconsistency can be attributed to insufficient sample sizes, heterogeneity in statistical methods, and biological complexity [19]. A pressing scientific challenge is to systematically integrate clinical features with multigene characteristics to construct robust, comprehensive predictive models.

The advancement of artificial intelligence and machine learning has opened new avenues for risk modeling in complex diseases [20,21]. Compared with conventional Cox regression models, machine learning approaches are better suited to handle high-dimensional data and to capture nonlinear relationships and higher-order interactions among variables [22,23]. In recent years, methods such as random survival forest (RSF), gradient boosting survival (GBSurvival), and fast survival support vector machine have been increasingly applied to prognostic studies across multiple cancer types, demonstrating superior discriminative ability and stability over traditional approaches [24,25]. At the same time, interpretability techniques such as Shapley Additive Explanations (SHAP) provide insights into the key determinants underlying model predictions, thereby enhancing their translational potential in clinical practice [26,27]. Although these strategies have been reported in breast cancer, lung cancer, and leukemia, systematic investigations into their use for dual prediction of mortality and relapse risk in patients with DLBCL remain scarce [24,28].

Existing research remains limited in several respects. Most studies focus primarily on OS in DLBCL, with insufficient attention to systematic assessment of relapse risk and a lack of dual-modeling frameworks capable of simultaneously predicting mortality and relapse outcomes [29,30]. In addition, many models are restricted to clinical variables or single molecular features, failing to achieve true integration of clinical information with transcriptomic data, thereby constraining their discriminative power and robustness [31,32]. More critically, methodological shortcomings in interpretability hinder the clinical acceptance and applicability of these models. Developing a machine learning model that integrates both clinical and molecular characteristics, enables concurrent prediction of mortality and relapse risk, and provides transparent interpretability is therefore of considerable clinical and scientific significance for the individualized management of DLBCL.

Building on these considerations, this study uses the large-scale DLBCL cohort from the publicly available GSE31312 database to integrate clinical characteristics with transcriptomic data and to construct and compare multiple survival prediction models, including FastSurvivalSVM, GBSurvival, and RSF. We first established a clinical baseline model using Cox regression and identified candidate genes closely associated with survival. We then systematically evaluated the performance of the machine learning models in predicting both mortality and relapse risk, while applying SHAP to dissect the contributions of key features and to elucidate the synergistic effects of clinical and molecular determinants in prognostic prediction. The proposed framework not only offers a robust multidimensional strategy for risk modeling but also provides new perspectives for individualized risk stratification, precision therapeutic decision-making, and the exploration of potential molecular mechanisms in DLBCL, underscoring its translational and clinical relevance.

## Methods

### Data Sources and Inclusion Criteria

Expression profile data from the GSE31312 cohort were obtained using the Affymetrix Human Genome U133 Plus 2.0 Array (GPL570). Raw cell expression language files were downloaded from the Gene Expression Omnibus database and uniformly preprocessed in the R/Bioconductor environment. Specifically, the robust multiarray average pipeline was applied to perform background correction, quantile normalization, and probe set-level summarization, yielding a gene expression matrix on the log₂ scale. Probe annotation was conducted using the official annotation package or files corresponding to GPL570 to map probe sets to gene symbols. Probe sets that could not be reliably mapped or that corresponded to multiple gene symbols were excluded from further analysis. For genes represented by multiple probe sets, a predefined “representative probe” selection strategy was used to ensure reproducibility and avoid selection bias. Within the training dataset, expression variability for each probe set across samples was calculated, and the probe set with the highest variability was selected as the representative probe for that gene. In sensitivity analyses, alternative probe-collapsing strategies—namely, taking the median or mean expression values across probe sets corresponding to the same gene—were also applied, and the results were confirmed to be consistent.

During data acquisition, the research team downloaded raw CEL files and corresponding clinical information from the Gene Expression Omnibus platform. All samples were cross-validated using unique patient identifiers to eliminate duplicate cases. The final cohort included cases with complete transcriptomic data, OS outcome information, and key clinical variables such as Age, ECOG score, LDH levels, Ann Arbor stage (AAS), B symptoms, and Gene Expression Profiling (GEP) subtype. OS was defined as the duration from diagnosis to death; for those still alive at last follow-up, data were censored accordingly.

In selecting study subjects, cases meeting the pathological diagnosis of DLBCL with comprehensive follow-up and core clinical information were included in the analysis; cases lacking key outcome indicators or core variables were excluded. Additionally, samples with significant outliers that could not be corrected upon review were also omitted from final analyses. These quality control measures ensured the scientific validity and reliability of data analysis. The study’s data are entirely based on publicly accessible databases; all analyses were conducted under ethical compliance and data-sharing principles. Relevant data and analytical procedures will be made publicly available post-publication to ensure transparency and reproducibility.

### Batch Effect Assessment and Correction

Quality control of the normalized expression matrix was performed using principal component analysis (PCA), hierarchical clustering, and density distribution plots, in conjunction with available technical metadata (eg, study center, batch, or processing date) to assess potential batch-driven clustering patterns. When pronounced batch effects were detected, ComBat (implemented in the sva package) was applied to the training dataset for batch correction, and the resulting adjustment parameters were subsequently transferred to the corresponding validation dataset. In cases where no substantial batch-driven structure was observed, batch correction was not enforced. Nevertheless, ComBat-based sensitivity analyses were conducted and reported to demonstrate the robustness of the study conclusions.

### Preprocessing of Clinical Variables

In the process of organizing clinical features, age is treated as a continuous variable (measured in years) and directly included in the analysis. The ECOG performance status score is retained in its original ordinal scale from 0 to 4 and entered into subsequent models as a continuous ordinal variable to reflect the linear trend of functional status. LDH levels are categorized into high and normal groups based on test results, processed as a binary variable. B symptoms are also dichotomized to capture their adverse impact on disease progression. The AAS system is set as an ordinal categorical variable and converted into numerical values (I=1, II=2, III=3, and IV=4) to ensure comparability in regression and machine learning models. The IPI, a well-established prognostic stratification tool, is directly input into the model in integer form, ranging from 0 to 5, for performance comparison with newly developed modeling tools.

At the molecular phenotype level, GEP subtypes are classified into Activated B-cell-like, Germinal Center B-cell-like, and unclassified (UC) categories based on previous classification standards, using one-hot encoding during modeling with UC as the reference class to ensure statistical interpretability. Other molecular markers, such as *CD10, BCL6, FOXP1,* and *MUM1*, are recorded continuously rather than dichotomously to retain detailed expression level information. The number of extranodal involvements (N_extran) is input as a count variable that can be used for continuous variable modeling or binned in sensitivity analyses (eg, 0, 1, and ≥2) to verify stability. All clinical variables underwent missing value detection prior to inclusion in the analysis; when missing rates were low, median or mode imputation was used, with sensitivity analyses conducted during model validation to assess the impact of imputation methods on outcomes. These preprocessing steps ensure consistency and robustness of clinical features in subsequent Cox regression and machine learning models.

### Transcriptomic Gene Screening

During the candidate gene screening process, transcriptomic data across the entire genome are initially prescreened using univariate Cox regression. This involves calculating each gene’s hazard ratio (HR), 95% CI, and raw *P* value, followed by ranking the candidate genes based on statistical significance. To enhance the robustness of the selection process, regularization selection is performed using Cox regression with an elastic net penalty (implemented via CoxnetSurvivalAnalysis from the scikit-survival package) before entering multivariable modeling. During parameter tuning, the l1_ratio is set at 1.0, 0.8, and 0.5, respectively, combined with alpha_min_ratio values of 0.1, 0.2, and 0.5 for grid search. Each parameter set undergoes performance evaluation under a 5-fold cross-validation framework, using Harrell C-index as an indicator of model discrimination ability. This process identifies the optimal combination of regularization parameters, with the corresponding average C-index serving as a benchmark to select the best-performing alpha value for subsequent modeling.

In the stability selection phase, bootstrap resampling is conducted 200 times; each time fitting a Coxnet model on the training set and recording the frequency at which each gene is selected in the model. Genes selected in more than 60% of iterations are considered core stable features, while those selected between 50% and 60% form a reserve set. If stability selection results are insufficient to construct a final feature set of 20 genes, penalty strength is reduced to refit the model to capture additional potential signals. Should gaps still exist, high-weight genes are supplemented according to absolute regression coefficients from the full model to ensure a complete feature set of 20 genes.

The final set of 20 genes integrates results from both univariate Cox regression screening and multivariable penalized regression stability selection while ensuring completeness and interpretability through a multitier fallback mechanism. The screening process was conducted in a Python environment using scikit-survival and scikit-learn libraries. All output results, including univariate Cox regression statistics tables, stability selection frequency tables, and the final gene set, are preserved to ensure transparency and reproducibility of the analytical workflow.

### Handling of Missing Data

Prior to inclusion in the analysis, a comprehensive assessment and documentation of missing values for all clinical and molecular characteristics were conducted. The proportion of missing data was explicitly reported, and its potential biasing effect on modeling was evaluated. For continuous variables, such as age and the number of extranodal sites, mean or median imputation was used when the missing proportion was low. Additionally, a missing indicator variable was added to capture potential information loss in cases where the missing proportion was relatively high or involved critical variables; multiple imputation by chained equations was used. This method generated several imputed datasets through iterative regression chains, and their estimates were combined in subsequent analyses to ensure robustness of the estimators. For categorical variables like B symptoms, LDH status, and GEP subtype, missing values were uniformly filled using the mode of the variable to minimize information bias due to random missingness. All imputation steps were completed before model fitting, and sensitivity analyses were conducted subsequently to examine the impact of different imputation strategies on result robustness.

### Construction and Evaluation of a Clinical Baseline Model

In constructing a clinical baseline model, univariate Cox proportional hazards regression analysis is initially performed on all candidate clinical and molecular variables, using OS and relapse-free survival (RFS) as endpoints. The HR, 95% CI values, and corresponding *P* values are calculated. Variables with *P*<.10 are selected for subsequent multivariate modeling, while certain recognized key factors, such as ECOG performance status and AAS, are forcibly included based on prior clinical knowledge.

In the multivariate Cox model, core variables ultimately included are age, LDH status, ECOG performance status, AAS, B symptoms, and GEP subtype. Immunophenotypic markers (*CD10, BCL6, FOXP1,* and *MUM1*) are also incorporated when necessary. During model fitting, the Schoenfeld residual test is used to verify the proportional hazards assumption, with sensitivity analysis through time interaction terms further validating model stability. If the proportional hazards assumption is violated, stratified Cox regression or time-dependent covariates are used for correction. To avoid model instability, all included variables undergo variance inflation factor testing, with variance inflation factor<5 set as the reference standard for the absence of severe multicollinearity. Model results are reported in terms of HRs, 95% CIs, and *P* values and visualized using forest plots on a logarithmic scale with T-shaped error bars indicating estimated CIs.

To further validate the clinical baseline model’s capacity for risk stratification, individualized risk scores are generated based on weighted regression coefficients and survival comparisons conducted using different grouping methods. In the dichotomous approach, patients are divided into high-risk and low-risk groups based on the median risk score; in the tertile approach, they are categorized into low-, medium-, and high-risk subgroups according to tertiles of risk scores. Survival differences among groups are illustrated using Kaplan-Meier survival curves and statistically evaluated via log-rank tests. This methodology systematically verifies the applicability and discriminative power of the clinical baseline model in prognostic assessment for patients with DLBCL.

### Gene-Level Association Analysis

To investigate the relationship between gene expression levels and patient prognosis, we selected 20 genes from a candidate set for individual analysis. Initially, we fitted a univariate Cox proportional hazards regression model for each gene, using OS and RFS as outcome measures. We calculated the HR, 95% CI, and corresponding *P* value for each gene. When necessary, we applied the Benjamini-Hochberg method to adjust for false discovery rate (FDR) to control for errors arising from multiple testing.

Subsequently, patients were stratified into high and low expression groups based on the median expression of each gene in the cohort. We used the Kaplan-Meier method to plot survival curves and used log-rank tests to evaluate differences between groups. This approach visually illustrates potential associations between gene expression levels and survival outcomes.

For visualization, all results from single-gene Cox analyses were presented as forest plots. These plots used a logarithmic scale with T-shaped error bars to display 95% CIs clearly, facilitating an understanding of effect sizes and their CIs. Additionally, Kaplan-Meier survival curves were presented in small multiples format, and where necessary, composite layouts were used to systematically display survival differences across varying expression levels of the 20 genes. This comprehensive analysis ensures that the gene-level association results are statistically robust and visually interpretable.

### Construction of Machine Learning Models

To enhance the accuracy of prognostic predictions, three types of machine learning-based survival models were developed: FastSurvivalSVM, GBSurvival, and RSF. Regarding feature inputs, all models integrate core clinical variables with the expression levels of candidate genes, ensuring a comprehensive consideration of both traditional prognostic factors and molecular-level information. To prevent data leakage, numerical variables were imputed and standardized using a column transformer during the modeling process. Categorical variables were binarized or one-hot encoded. The preprocessing and modeling stages were connected through a pipeline structure to achieve full automation and consistency throughout the process.

### Cross-Validation Framework and Control of Information Leakage

To prevent potential information leakage during high-dimensional transcriptomic feature selection and model training, a nested 5-fold cross-validation framework was implemented. The outer loop was used for independent evaluation of model performance, in which one fold was held out as the outer validation set while the remaining four folds constituted the outer training set. The outer validation fold was strictly excluded from all gene selection procedures, stability selection, and hyperparameter tuning within that iteration. Within each outer training set, the complete internal modeling workflow was repeated in the inner loop, including: (1) genome-wide univariate Cox regression–based prescreening conducted exclusively within the training folds; (2) regularized feature selection and parameter optimization using elastic net-penalized Cox regression (Coxnet); (3) bootstrap-based stability selection to identify a stable set of prognostic genes; and (4) training and hyperparameter tuning of FastSurvivalSVM, gradient boosting survival models (GBSurvival), and random survival forest models. For the FastSurvivalSVM model, calibration of the risk score direction (decision margin direction) was also performed solely within the training folds and subsequently fixed and applied to the corresponding outer validation fold. Through this strictly nested procedure, all steps, from feature discovery and parameter optimization to model fitting, were confined to the training data only, thereby effectively preventing information leakage and ensuring objective and reliable performance evaluation.

### Cross-Validation and Parameter Tuning

Model training and hyperparameter optimization were performed within a nested 5-fold cross-validation framework. The outer cross-validation loop was used for model performance evaluation, whereas the inner cross-validation loop was used for feature selection, hyperparameter tuning, and model training. Parameter tuning used a random search strategy (ParameterSampler), evaluating combinations drawn from a predefined candidate parameter space. For FastSurvivalSVM, the primary search focused on an alpha range from 1×10⁻⁵ to 1×10² (log-uniform distribution), with rank_ratio set at 0.2, 0.5, and 1.0. For GBSurvival, the search space included learning_rate (0.01–0.2), max_depth (1-3), n_estimators (200-1000), and subsample (0.6-1.0). For RSF, parameters ranged across n_estimators (500-1500), max_features (√p, log₂(p), or 0.3-0.8), and min_samples_leaf (1-10). Within each training fold, risk direction was further examined; if the model’s risk scores contradicted the actual event direction, margin direction was adjusted accordingly and consistently applied during validation folds and full data training phases.

### Model Evaluation Metrics

In terms of performance evaluation, Harrell C-index is initially used to assess the model’s discriminative ability, with results reported for each fold along with the median and IQR values. Subsequently, cumulative_dynamic_auc is used to calculate the time-dependent receiver operating characteristic (ROC) curves and corresponding area under the curve (AUC) values at 1-year, 3-year, and 5-year intervals. CIs are estimated using the bootstrap method (≥1000 resamples). Calibration assessment is based on deciles or groupings of risk scores, comparing predicted probabilities with Kaplan-Meier observed rates at target time points. Calibration curves are plotted, and the calibration slope and intercept are calculated. Clinical benefit is evaluated through decision curve analysis (DCA), comparing the net benefit of the model across different threshold probabilities against “treat none” and “treat all” strategies. The model’s risk stratification capability is visually demonstrated through risk score distributions and survival status strip charts. Additionally, Kaplan-Meier curves are plotted based on dichotomous and trichotomous methods, with log-rank tests used to assess differences between groups.

### Interpretability Analysis Using SHAP

In terms of interpretability, FastSurvivalSVM is selected as the primary subject of analysis. The SVM decision function margin serves as the target for interpretation, and the Kernel SHAP method is used to approximate the contribution of each feature to risk prediction. To ensure robustness, 100 samples are randomly selected from the entire dataset as a background set for computation. Sensitivity analysis involves adjusting the background set size (ranging from 50 to 200) to test stability. The output includes global feature importance bar charts (featuring the top-ranked features by mean |SHAP|) and beeswarm plots, which visually depict the relationship between feature values and the direction of risk prediction. By integrating these interpretative results, we further elucidate the synergistic contribution patterns of clinical features (such as IPI score, AAS, age, ECOG score, and number of extranodal involvements) and molecular features (such as *PSMG4*, *CRY1*, *LENEP*, *SPIRE1*, *CKS1B*, and *TMEM182*; Figure S1 in Multimedia Appendix 1).

### Statistical Analysis

All statistical tests were conducted as 2-sided tests, with a significance threshold set at *P*<.05. When necessary, the FDR was adjusted using the Benjamini-Hochberg method. CIs for all estimates were based on 95% CIs, and those for AUC and C-index were calculated via bootstrapping (≥1000 iterations). Data analysis and model construction were performed in a Python environment (versions 3.9/3.10), using key libraries such as lifelines, scikit-survival, scikit-learn, pandas, numpy, matplotlib, and shap. Supplementary analyses and graphical representations were completed in an R environment (R Core Team) using the survival and survminer packages. To ensure reproducibility, a fixed random seed was set throughout the analysis process, with details of the computational environment and software versions documented.

### Ethical Considerations

This research constitutes a secondary analysis based on public databases, fully adhering to database usage terms and the ethical principles of the Declaration of Helsinki, thus not requiring additional ethical approval.

This study is a secondary analysis based on publicly available data from the Gene Expression Omnibus (GEO) database (accession number: GSE31312). All data used in this study are de-identified and publicly accessible, and therefore do not involve direct human participant interaction or identifiable personal information. According to institutional and international guidelines, including the Declaration of Helsinki, research based on publicly available, anonymized datasets does not require formal approval from an institutional review board or ethics committee. Therefore, ethics approval and informed consent were not required for this study. All procedures were conducted in accordance with relevant data usage policies and regulations.

## Results

### Application of Clinical Baseline Models in Mortality Risk Prediction

The clinical characteristics of the included patients are summarized in Multimedia Appendix 2.

**Figure 1:**
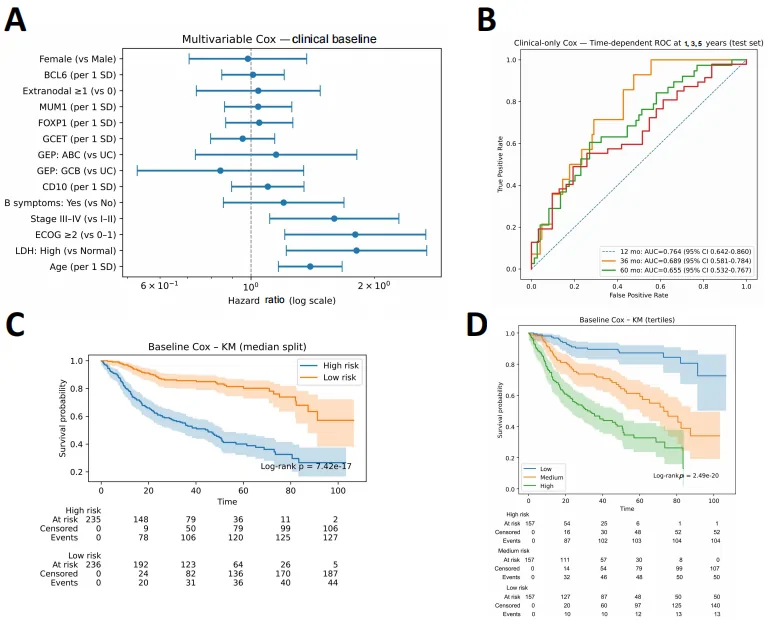
Prognostic performance of the clinical baseline model in overall survival prediction. (A) Multivariate Cox regression forest plot identifies age, lactate dehydrogenase elevation, Eastern Cooperative Oncology Group performance status ≥2, and Ann Arbor stage III-IV as independent prognostic factors. (B) Time-dependent receiver operating characteristic curves demonstrate area under the curve values of 0.764, 0.689, and 0.655 at 1, 3, and 5 years, respectively. (C) Kaplan-Meier curves stratified by median risk score show significantly reduced survival in the high-risk group. (D) Kaplan-Meier curves stratified by tertiles of risk score reveal a clear stepwise separation of survival outcomes. BCL6: B-cell lymphoma 6; ECOG: Eastern Cooperative Oncology Group; FOXP1: Forkhead box protein P1; GCET: Germinal Center Expressed Transcript; GEP:ABC: Gene expression profiling activated B-cell like; GEP:GBC: Gene expression profiling germinal center B like; KM: Kaplan-Meier; LDH: lactate dehydrogenase; MUM1: multiple myeloma oncogene 1; ROC: receiver operating characteristic; UC: unclassified.

To evaluate the prognostic predictive capacity of clinical features, we initially developed a clinical baseline model based on multivariate Cox regression. The results indicated that factors such as age, elevated LDH, an ECOG score of ≥2, and AAS III-IV were closely associated with poor survival outcomes (Figure 1A; Multimedia Appendix 3). The model achieved a C-index of 0.674 in the overall cohort, suggesting a moderate level of discrimination for patient mortality risk. Further time-dependent ROC analysis demonstrated that the model had an AUC of 0.764 (95% CI 0.642-0.860) at 1 year, 0.689 (95% CI 0.581**-**0.784) at 3 years, and 0.655 (95% CI 0.532**-**0.767) at 5 years (Figure 1B), indicating better performance in short-term prognostic prediction, with a decline in long-term predictive ability. In the OS analysis, a total of 471 patients with DLBCL were included. Among them, 167 out of 471 (35.5%) patients experienced events, while 304 out of 471 (64.5%) patients were censored at the end of follow-up due to the absence of observed events. The median follow-up duration was 35 (IQR 17.3-56.7) months. Meanwhile, Kaplan-Meier analysis showed that the model effectively differentiated patient survival differences whether risk scores were dichotomized or trichotomized. Patients in the high-risk group exhibited a significantly lower OS rate compared to the low-risk group, and the survival curves of the low, intermediate, and high-risk groups displayed a clear stepwise distribution, with statistically significant differences (log-rank *P*<.001; Figure 1C and D).

In summary, the clinical baseline model can effectively predict the survival risk of patients with DLBCL, providing a reference for subsequent integrative analysis of molecular characteristics.

### Association Analysis of Genomic-Level Mortality Risk Prediction

Building upon genome-wide Cox regression screening, we further conducted univariate Cox regression analysis on 20 candidate genes. To facilitate comprehension of the gene selection and feature construction process, we supplemented the study with a schematic diagram. This diagram visually illustrates the step-by-step construction process from genome-wide screening, univariate Cox regression, stability selection, to the final 20-gene feature set (Figure S2A in Multimedia Appendix 1 and Multimedia Appendix 4). The results indicate that alterations in transcription levels of most genes are significantly associated with patients’ OS. Specifically, genes such as *CERS6*, *HIC1*, *CRAT*, *COPE*, *CTBP2*, *MT2A*, *GBP2*, *ABCD2*, *INPP4B*, *BBS9*, *KPNA5*, *AAK1*, *FYB*, *HHEX*, *CASC9*, *CRY1*, *PSMG4*, and *CRTAM* exhibit consistent risk effects in the model, with some HR significantly deviating from 1, suggesting their potential independent prognostic value (Figure S2B in Multimedia Appendix 1).

Further Kaplan-Meier survival analysis aligns with the Cox regression findings. Patients with higher expression levels of genes such as *PSMG4*, *CRY1*, *CRTAM*, *CTBP2*, *HHEX*, and *CASC9* demonstrate notably poorer survival outcomes, with survival curves distinctly separating (Figure S2C in Multimedia Appendix 1). For instance, the median survival of the high-expression group of *PSMG4* is markedly reduced, and similarly, high expression of *CRY1* and *CRTAM* indicates adverse prognosis. Conversely, some genes, such as *CERS6*, *FYB*, and *KPNA5*, although statistically significant in univariate Cox regression, show weaker separation in their Kaplan-Meier curves. Overall, these findings suggest that the aforementioned genes may play a crucial role in mortality risk among patients with DLBCL and provide a potential molecular basis for subsequent multivariate modeling.

### Comparative Analysis of Predictive Performance in Mortality Risk Forecasting Machine Learning Models

**Figure 2:**
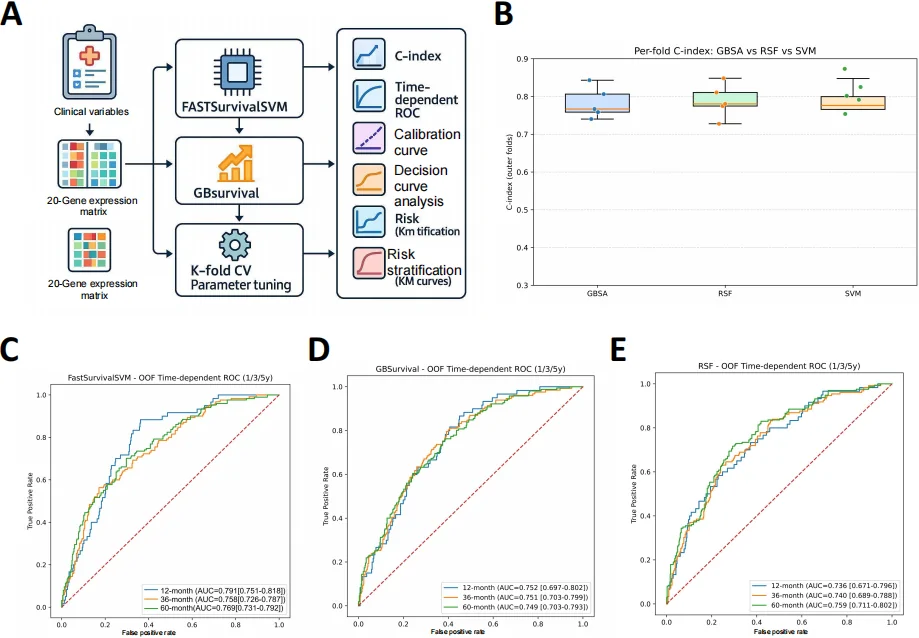
Benchmark comparison of three machine learning survival models for OS prediction. (A) Workflow schematic of machine learning modeling integrating clinical and transcriptomic features. (B) Distribution of C-index values for GBSurvival, random survival forest, and FastSurvivalSVM, all approximating 0.7. (C) Time-dependent receiver operating characteristic curves of the FastSurvivalSVM yield area under the curve values of 0.791, 0.758, and 0.769 at 1, 3, and 5 years. (D) Time-dependent receiver operating characteristic curves of the GBSurvival model show area under the curve values of 0.752, 0.751, and 0.749 at 1, 3, and 5 years. (E) Time-dependent receiver operating characteristic curves of the random survival forest model present area under the curve values of 0.736, 0.740, and 0.759 at 1, 3, and 5 years. FastSurvivalSVM: fast survival support vector machine; GBSurvival: gradient boosting survival; GBSA: Gradient Boosting Survival Analysis; RSF: random survival forest; SVM: support vector machine; ROC: receiver operating characteristic; OOF: Out-of-Fold.

To enhance the accuracy of prognostic predictions, we developed three machine learning survival models based on clinical and genetic features: FastSurvivalSVM, GBSurvival, and RSF. During the external validation by 5-fold cross-validation, all 3 models demonstrated stable predictive capabilities, albeit with varying degrees of discrimination. To clearly present the modeling approach and evaluation process, we supplemented our study with a flowchart systematically illustrating the entire process, from the input of clinical and transcriptomic data, model construction, and parameter tuning to cross-validation and performance assessment (Figure 2A).

The results from the 5-fold cross-validation indicated that the C-index distributions of the three models were similar, all maintaining around a level of 0.7, suggesting comparable discrimination in patient survival risk (Figure 2B). Time-dependent ROC analysis further corroborated these findings. The FastSurvivalSVM model achieved AUCs of 0.791 (95% CI 0.751**-**0.818), 0.758 (95% CI 0.726**-**0.787), and 0.769 (95% CI 0.731**-**0.792) for 1-year, 3-year, and 5-year predictions, respectively, indicating high stability in both short-term and long-term forecasts (Figure 2C). In comparison, the GBSurvival model yielded AUCs of 0.752 (95% CI 0.697**-**0.802), 0.751 (95% CI 0.703**-**0.799), and 0.749 (95% CI 0.703**-**0.793) for the same periods, showing similar but slightly lower performance (Figure 2D); the RSF model presented AUCs of 0.736 (95% CI 0.671**-**0.796), 0.740 (95% CI 0.689**-**0.788), and 0.759 (95% CI 0.711**-**0.802), with overall predictive performance akin to GBSurvival but marginally inferior to FastSurvivalSVM in short-term predictions (Figure 2E).

In conclusion, all three machine learning models effectively differentiated high-risk and low-risk groups among patients with DLBCL, with the FastSurvivalSVM model exhibiting the best overall performance. Consequently, it was selected as the primary modeling tool for subsequent analyses.

### Clinical Predictive Value of the FastSurvivalSVM Model for Mortality Risk Prediction

**Figure 3:**
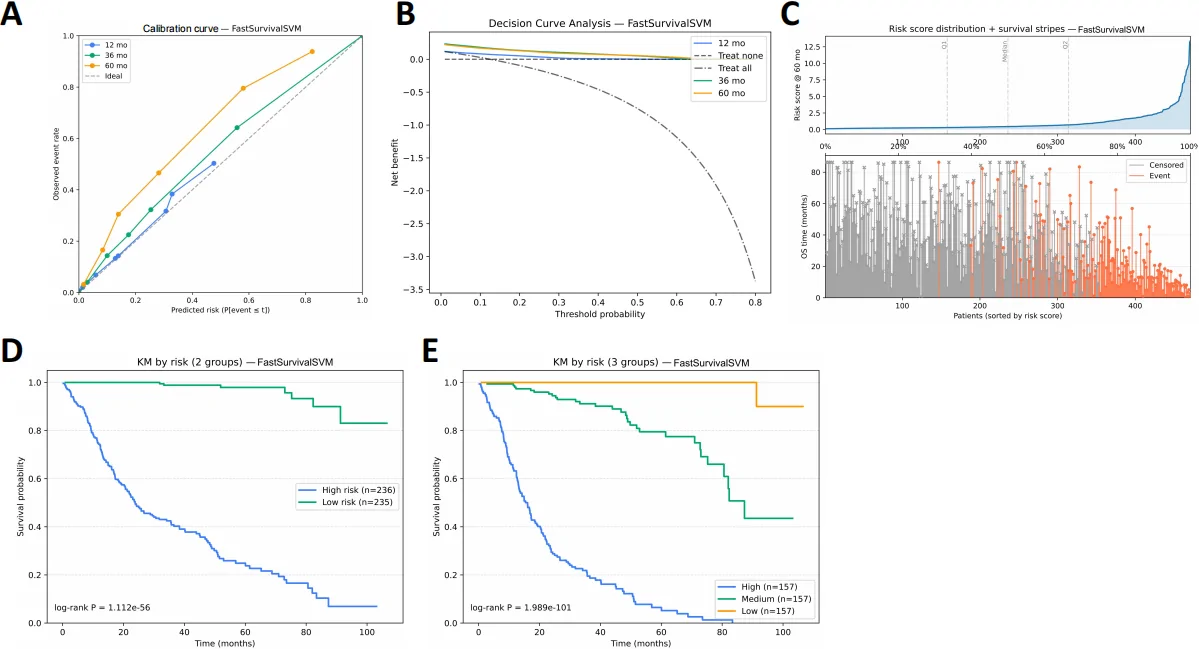
Clinical use and risk stratification performance of the fast survival support vector machine model for overall survival. (A) Calibration curves at 1, 3, and 5 years demonstrate good agreement between predicted and observed survival. (B) Decision-curve analysis shows consistently higher net benefit of the model compared with treat-none and treat-all strategies across a range of threshold probabilities. (C) Risk score distribution and survival status plots indicate that mortality events are concentrated in the high-risk group. (D) Kaplan-Meier curves stratified by median risk score reveal significantly reduced survival in the high-risk cohort. (E) Kaplan-Meier curves stratified by tertiles of risk score display a clear stepwise separation of low-, intermediate-, and high-risk groups. KM: Kaplan-Meier; OS: overall survival.

In further evaluating the clinical applicability of the FastSurvivalSVM model, calibration curves indicate that the model exhibits excellent fit at 1-year, 3-year, and 5-year intervals. The predicted probabilities are highly consistent with the actual observed event rates, suggesting robust reliability of the model (Figure 3A). DCA further demonstrates that at various threshold probabilities, the model consistently provides high net benefits, showing a significant advantage over the “treat none” and “treat all” strategies (Figure 3B).

The distribution of risk scores and survival status strip charts reveals that as risk scores increase, mortality events are significantly concentrated at the high-risk end, indicating the effectiveness of the model’s risk stratification (Figure 3C). Kaplan-Meier survival analysis further corroborates this finding. Grouping based on median scores shows notably lower OS rates in the high-risk group compared with the low-risk group (Figure 3D), while tertile grouping similarly exhibits a stepwise survival difference among low, medium, and high-risk groups, further validating the model’s robustness in risk stratification (Figure 3E).

In conclusion, the FastSurvivalSVM model not only demonstrates stable overall predictive performance but also achieves excellent calibration and clinical decision value. It effectively distinguishes between different risk levels, providing a reliable tool for individualized prognosis assessment in DLBCL.

### Supplementary Validation of GBSurvival and RSF Models for Mortality Risk Prediction

To further assess the predictive value of other machine learning models, we conducted an independent validation of the GBSurvival and RSF models. Calibration curves demonstrated that the predictions made by both models at 1, 3, and 5 years aligned closely with the actual observed outcomes, indicating good calibration (Figure S3A and S3B in Multimedia Appendix 1). DCA further illustrated that both GBSurvival and RSF models provided higher net benefits across various threshold probabilities compared to the “treat none” and “treat all” strategies, thereby affirming their potential use in clinical decision-making (Figure S3C and S3D in Multimedia Appendix 1).

The distribution of risk scores and survival status strip charts further elucidated the models’ performance in patient stratification. The RSF model distinguished clearly between high and low-risk patients, whereas the GBSurvival model, although capable of identifying clusters of high-risk events, showed relatively weaker overall differentiation (Figure S4A and S4B in Multimedia Appendix 1). Kaplan-Meier analysis results supported this conclusion. The GBSurvival model effectively differentiated patient survival outcomes in both binary and tertiary groupings (Figure S4C and S4D in Multimedia Appendix 1); similarly, the RSF model demonstrated significant stepwise survival differences in these stratifications, underscoring its reliability in risk stratification (Figure S4E and S4F in Multimedia Appendix 1).

In summary, although the overall predictive performance of GBSurvival and RSF models shows limited differences with the FastSurvivalSVM model, they exhibit commendable calibration, clinical benefit, and risk stratification capabilities. These models offer diversified modeling options for individualized prognostic assessment of patients with DLBCL.

### Analysis of Feature Importance and Interpretability in FastSurvivalSVM Model for Mortality Risk Prediction

**Figure 4:**
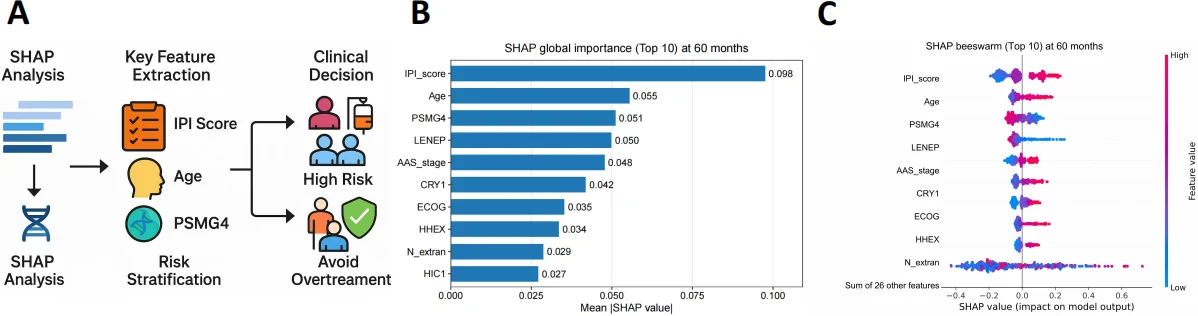
Model interpretability and key prognostic features identified by fast survival support vector machine for overall survival. (A) Schematic representation of Shapley Additive Explanations analysis for feature extraction and potential clinical translation. (B) Shapley Additive Explanations summary bar plot identifies the top 10 features, including international prognostic index score, age, PSMG4, LENEP, Ann Arbor stage, CRY1, Eastern Cooperative Oncology Group, HHEX, number of extranodal sites, and HIC1. (C) Beeswarm plot illustrates that high expression of PSMG4, CRY1, and LENEP is associated with poor outcomes, whereas low international prognostic index scores and younger age indicate favorable prognosis. AAS: Ann Arbor stage; ECOG: Eastern Cooperative Oncology Group; IPI: international prognostic index; SHAP: Shapley Additive Explanations.

This study uses SHAP methodology to elucidate the predictive basis of the FastSurvivalSVM model, focusing on feature importance and interpretability analysis. The aim is to identify key clinical and molecular features that significantly influence model predictions. To illustrate how the model’s interpretative outcomes can be applied in clinical practice, we provide a process diagram that visually outlines the entire procedure–from extracting critical features via SHAP analysis to achieving patient risk stratification, ultimately guiding clinical decision-making (Figure 4A). Both clinical and molecular features play pivotal roles in the model’s prediction, with IPI score, age, *PSMG4*, *LENEP*, AAS_stage, *CRY1*, ECOG score, *HHEX*, N_extran, and *HIC1* being among the top 10 contributing features.

The feature importance bar chart indicates that IPI score and age are ranked highest in terms of overall contribution, underscoring the central role of clinical indicators in assessing the prognosis of DLBCL. Concurrently, molecular features such as *PSMG4*, *LENEP*, and *CRY1* hold significant weight in the model, suggesting that abnormal molecular expression is closely related to patient survival risk (Figure 4B). The beeswarm plot further reveals how different feature values influence the direction of risk prediction: higher expression of *PSMG4*, *CRY1*, and *LENEP* is associated with poor outcomes, whereas lower IPI scores and younger patients correlate with better survival outcomes (Figure 4C).

In summary, the SurvivalSVM model effectively integrates clinical and molecular features, identifying the most influential variables for prognosis prediction. SHAP interpretability analysis provides transparency in model decision-making, offering a reliable foundation for risk stratification and mechanism understanding in patients with DLBCL.

### Application of Clinical Baseline Model in Recurrence Risk Prediction

**Figure 5:**
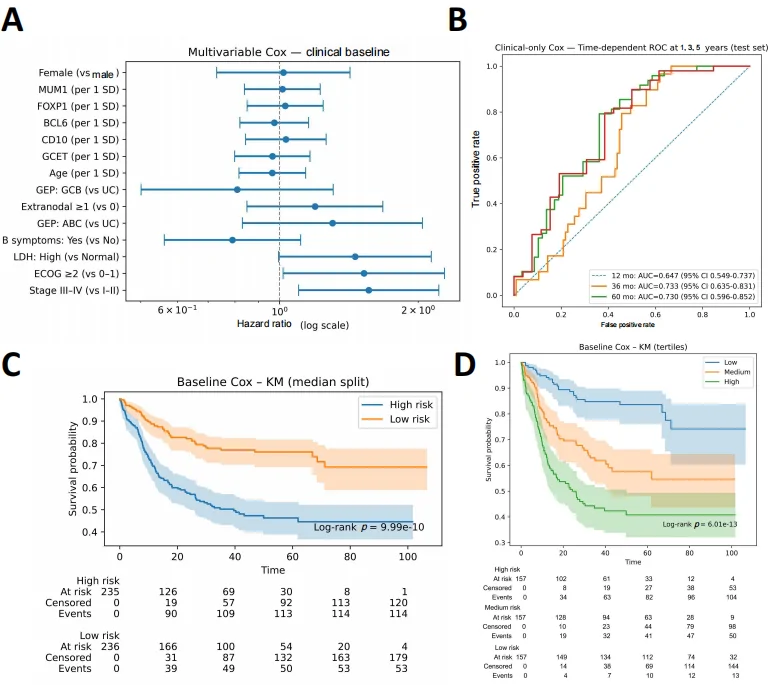
Prognostic performance of the clinical baseline model in random forest survival prediction. (A) Multivariate Cox regression forest plot identifies Ann Arbor stage, Eastern Cooperative Oncology Group performance status ≥2, elevated lactate dehydrogenase, B symptoms, and gene expression profiling subtype as independent prognostic factors. (B) Time-dependent receiver operating characteristic curves show area under the curve values of 0.647, 0.733, and 0.730 at 1, 3, and 5 years; (C) Kaplan-Meier curves stratified by median risk score reveal significantly higher relapse rates in the high-risk group. (D) Kaplan-Meier curves stratified by tertiles of risk score demonstrate a clear stepwise separation among low-, intermediate-, and high-risk groups. BCL6: B-cell lymphoma 6; ECOG: Eastern Cooperative Oncology Group; FOXP1: Forkhead box protein P1; GCET: Germinal Center Expressed Transcript; GEP:ABC: Gene expression profiling activated B-cell like; GEP:GBC: Gene expression profiling germinal center B like; KM: Kaplan-Meier; LDH: lactate dehydrogenase; MUM1: multiple myeloma oncogene 1; ROC: receiver operating characteristic; UC: unclassified.

In the context of recurrence risk prediction, we initially developed a clinical baseline model based on a multivariate Cox regression analysis. The results indicated a significant association between recurrence risk and factors such as AAS III-IV, an ECOG score of ≥2, and elevated LDH levels (Figure 5A; Multimedia Appendix 5). The overall model demonstrated a C-index of 0.653, indicating a moderate discriminative ability in predicting patient recurrence risk. Time-dependent ROC analysis revealed that the model’s AUC for predictions at 1, 3, and 5 years were 0.647 (95% CI 0.549**-**0.737), 0.733 (95% CI 0.635**-**0.831), and 0.730 (95% CI 0.596**-**0.852), respectively (Figure 5B), suggesting stable performance in long-term recurrence risk prediction.

Further validation of the clinical baseline model’s risk stratification capability was conducted through Kaplan-Meier survival analysis. Median-based dichotomous analysis demonstrated a significantly higher recurrence rate in high-risk patients compared to low-risk patients (Figure 5C). Meanwhile, in the tertile analysis, the survival curves for low, medium, and high-risk groups exhibited a clear stepwise distribution, with differences being highly statistically significant (Figure 5D).

In summary, the clinical baseline model provides a degree of differentiation in recurrence risk among patients with DLBCL, offering a reference point for subsequent integration with molecular characteristics and machine learning modeling.

### Association Analysis of Gene-Level Recurrence Risk Prediction

Based on a comprehensive genome-wide screening, we conducted univariate Cox regression analysis on 20 candidate genes to evaluate their independent prognostic value in predicting recurrence risk. The findings revealed that the expression levels of certain genes were significantly associated with recurrence risk. Specifically, the HR of genes such as *TMEM182*, *SPIRE1*, *CKS1B*, *MIR30D*, *MDK*, *DLEU1*, *MIR532*, and *ARIH2* were significantly divergent from 1, suggesting their potential independent prognostic role in recurrence event prediction (Figure S5A; Multimedia Appendix 6).

Further validation through Kaplan-Meier analysis confirmed these observations. High expression levels of genes such as *TMEM182*, *SPIRE1*, MIR30D, *CKS1B*, and *MDK* were associated with shorter RFS times (log-rank *P*<.05), with survival curves clearly separating between high and low expression groups. In contrast, although the Cox analysis indicated potential effects for some genes like *IBSP*, *NLRP11*, *DIP2A*, and *EFS*, no significant differences were evident in their survival curves (Figure S5B in Multimedia Appendix 1).

In summary, these findings indicate that numerous molecular characteristics play pivotal roles in recurrence risk, with several key genes potentially acting as driving factors in the progression of DLBCL recurrence. These insights provide candidate features for the development of comprehensive predictive models.

### Comparative Performance Analysis of Multiple Models in Recurrence Risk Prediction

**Figure 6:**
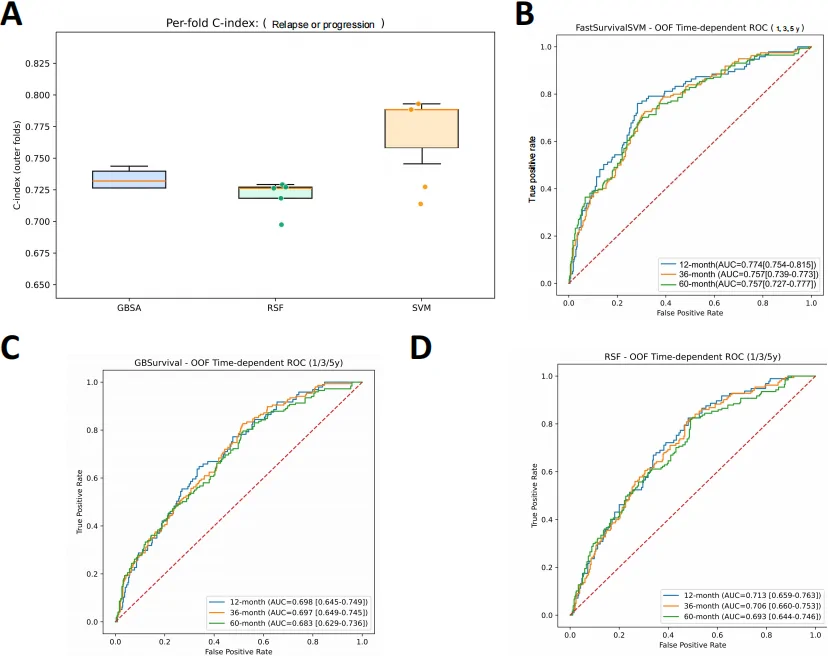
Comparative performance of three machine learning models in relapse-free survival prediction. (A) The distributions of C-index values for the three models are comparable, ranging from 0.70 to 0.78. (B) The fast survival support vector machine model achieves area under the curve values of 0.774, 0.757, and 0.757 at 1, 3, and 5 years, respectively. (C) The corresponding area under the curve values of the random survival forest model are 0.713, 0.706, and 0.693 at 1, 3, and 5 years. (D) The gradient boosting survival model yields area under the curve values of 0.698, 0.697, and 0.683 at 1, 3, and 5 years. GBSA: Gradient Boosting Survival Analysis; RSF: random survival forest; SVM: support vector machine; OOF: Out-of-Fold; ROC: receiver operating characteristic; FastSurvivalSVM: fast survival support vector machine; GBSurvival: gradient boosting survival.

In the context of recurrence risk prediction, we conducted a comparative analysis of the predictive performance of three machine learning models: GBSurvival, RSF, and FastSurvivalSVM. During cross-validation for recurrence risk prediction, all three models demonstrated relatively stable discrimination capabilities. The C-index distribution for GBSurvival, RSF, and FastSurvivalSVM was generally concentrated between 0.70 and 0.78, with median performances across different folds showing no significant advantage for any model (Figure 6A). This finding indicates that all three models consistently provide robust discrimination in recurrence or progression risk prediction.

Time-dependent ROC analysis further elucidated the predictive performance of different models at various time points. The FastSurvivalSVM model achieved AUC values of 0.774 (95% CI 0.754**-**0.815), 0.757 (95% CI 0.739**-**0.773), and 0.757 (95% CI 0.727**-**0.777) at 1, 3, and 5 years, respectively, indicating stable overall performance (Figure 6B). The RSF model’s AUCs at the corresponding time points were 0.713 (95% CI 0.659**-**0.763), 0.706 (95% CI 0.660**-**0.753), and 0.693 (95% CI 0.644**-**0.746), suggesting a short-term predictive advantage (Figure 6C). In contrast, the AUCs for the GBSurvival model were 0.698 (95% CI 0.645**-**0.749), 0.697 (95% CI 0.649**-**0.745), and 0.683 (95% CI 0.629**-**0.736), slightly lower overall compared to the other two methods (Figure 6D).

In summary, all three machine learning models effectively predict the recurrence risk of DLBCL, with FastSurvivalSVM exhibiting superior overall stability, while the RSF model shows certain competitive strengths in short-term predictions.

### Validation of the Clinical Use of the FastSurvivalSVM Model in Predicting Recurrence Risk

**Figure 7:**
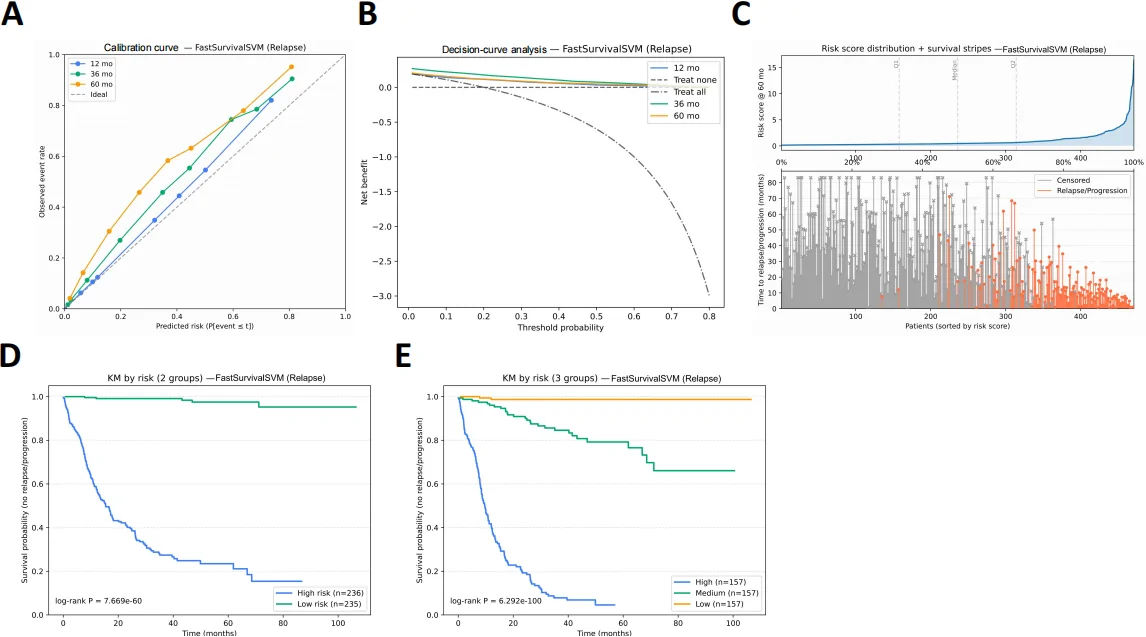
Clinical use and risk stratification of the fast survival support vector machine model for relapse-free survival. (A) Calibration curves at 1, 3, and 5 years show close agreement between predicted and observed outcomes. (B) Decision-curve analysis demonstrates clear net benefit across a wide range of threshold probabilities. (C) Risk score distribution and survival status plots indicate that relapse events cluster at the high-risk end. (D) Kaplan-Meier curves stratified by median risk score reveal significantly higher relapse rates in the high-risk group. (E) Kaplan-Meier curves stratified by tertiles of risk score display highly significant stepwise separation among low-, intermediate-, and high-risk groups.

Upon further evaluation of the clinical applicability of the FastSurvivalSVM model in predicting recurrence risk, the calibration curve results indicate a high concordance between the model’s predicted probabilities at 1, 3, and 5 years and the observed recurrence rates, demonstrating excellent predictive reliability and calibration (Figure 7A). DCA further reveals that across different threshold probabilities, the net benefit of using this model surpasses both the “treat none” and “treat all” strategies, highlighting its significant advantage in clinical decision-making (Figure 7B).

The distribution of risk scores and survival status stripes shows that recurrence events are markedly concentrated in patients with high-risk scores, suggesting that the model effectively identifies high-risk subgroups (Figure 7C). Kaplan-Meier survival analysis further corroborates the model’s risk stratification capability. Results from median-based dichotomization indicate that the recurrence and progression probability is significantly higher in the high-risk group compared to the low-risk group (log-rank *P*=7.67×10⁻⁶⁰; Figure 7D). Additionally, the tercile analysis reveals a pronounced stepwise difference among low, intermediate, and high-risk patients, with extremely high statistical significance (log-rank *P*=6.29×10⁻¹⁰⁰; Figure 7E).

In summary, the FastSurvivalSVM model not only demonstrates robust overall predictive accuracy but also exhibits superior calibration, clinical decision value, and risk stratification capabilities, providing a powerful tool for assessing recurrence risk and facilitating personalized management in patients with DLBCL.

### Supplementary Validation of GBSurvival and RSF Models in Recurrence Risk Prediction

In the context of recurrence risk prediction, both GBSurvival and RSF models demonstrate commendable predictive capabilities. Calibration curves indicate that the predicted probabilities at 1, 3, and 5 years align closely with the observed recurrence rates, suggesting robust model fit and calibration (Figure S6A and S6B in Multimedia Appendix 1). DCA further reveals that both models yield a higher net benefit across various threshold probabilities compared to the “treat none” and “treat all” strategies, confirming their potential clinical applicability (Figure S6C and S6D in Multimedia Appendix 1).

The distribution of recurrence risk scores and survival status strip charts further elucidates the models’ stratification performance. The RSF model achieves clear separation between high- and low-risk patients, while the GBSurvival model, although capable of identifying clusters of high-risk events, exhibits relatively weaker overall discrimination (Figure S7A and S7B in Multimedia Appendix 1). Kaplan-Meier analysis corroborates this conclusion. The GBSurvival model effectively distinguishes patients’ recurrence OR progression risk in both binary and ternary groupings, with log-rank test results of *P*=9.32×10⁻²⁵ and *P*=1.74×10⁻³⁶, respectively (Figure S7C and S7D in Multimedia Appendix 1). The RSF model demonstrates even more pronounced survival curve separation in its binary and ternary groupings (log-rank *P*=5.11×10⁻⁶⁶ and *P*=1.99×10⁻¹³⁸), with differences reaching high statistical significance (Figure S7E and S7F in Multimedia Appendix 1).

In summary, both GBSurvival and RSF models exhibit excellent calibration, clinical benefit, and stratification capabilities in recurrence risk prediction. They complement the FastSurvivalSVM model, collectively underscoring the value of integrating clinical and molecular characteristics in DLBCL recurrence risk assessment.

### Feature Importance and Interpretability Analysis of FastSurvivalSVM Model in Recurrence Risk Prediction

To further elucidate the decision-making basis of the FastSurvivalSVM model in predicting recurrence risk, we conducted an analysis of feature importance and interpretability using the SHAP method. The findings indicate that both clinical variables and molecular features play pivotal roles in the model’s predictions. Among the top 10 contributing features are AAS, IPI score, *XPA*, *ARIH2*, *DPY19L1P1*, *SPIRE1*, *EFS*, *CKS1B*, *TMEM182*, and N_extran.

**Figure 8:**
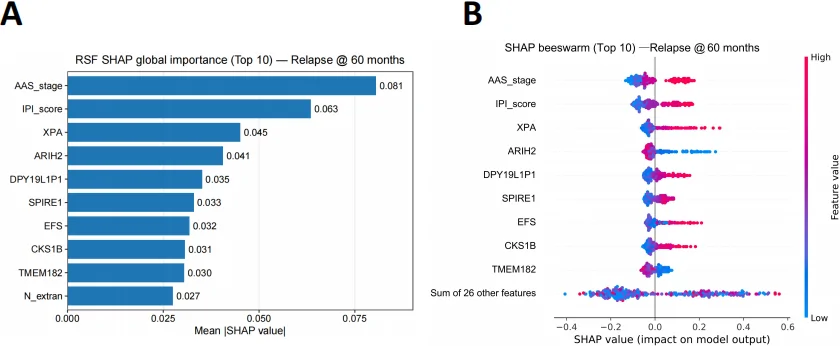
Shapley Additive Explanations–based interpretability analysis of the FastSurvivalSVM model for relapse-free survival. (A) Shapley Additive Explanations summary bar plot identifies Ann Arbor stage, international prognostic index score, XPA, ARIH2, DPY19L1P1, SPIRE1, EFS, CKS1B, TMEM182, and number of extranodal sites as the top 10 contributing features. (B) Shapley Additive Explanations beeswarm plot shows that high Ann Arbor stage, international prognostic index score, SPIRE1, CKS1B, and TMEM182 expression are associated with unfavorable relapse risk, whereas lower clinical scores indicate better prognosis.

The bar chart of feature importance demonstrates that AAS stage and IPI score hold the highest weights in model predictions, underscoring the central role of clinical characteristics in stratifying recurrence risk. Concurrently, molecular features such as the genes *XPA*, *ARIH2*, *SPIRE1*, *CKS1B*, and *TMEM182* also exhibit significant contributions, suggesting their potential driving role in the recurrence mechanisms of DLBCL (Figure 8A). The beeswarm plot further reveals the relationship between feature values and the direction of model predictions: higher AAS stage, IPI score, and elevated expression of *SPIRE1*, *CKS1B*, and *TMEM182* are closely associated with adverse recurrence outcomes, while lower clinical scores or feature levels suggest better event-free survival (Figure 8B).

In summary, the SHAP analysis of the FastSurvivalSVM model indicates that the model effectively captures the primary drivers of recurrence risk, highlighting the synergistic interaction between clinical features and key molecular markers. This provides crucial insights for the precise prediction of DLBCL recurrence risk and the exploration of potential biological mechanisms.

## Discussion

This study systematically evaluated the role of clinical and transcriptomic characteristics in prognostic prediction for DLBCL and developed various machine learning models to simultaneously predict patient mortality and recurrence risk. Through a comprehensive comparison of methods such as FastSurvivalSVM, GBSurvival, and RSF, we found that FastSurvivalSVM demonstrated superior performance in terms of model discrimination, calibration, and stability, particularly maintaining consistent accuracy in both short-term and long-term predictions. Notably, this study not only confirmed the value of clinical baseline models in prognostic assessment but also further revealed the complementary significance of molecular characteristics in risk stratification. Through SHAP interpretability analysis, the synergistic role of clinical and molecular factors within the model was elucidated. These findings provide a more comprehensive and transparent modeling framework for risk management in DLBCL.

The model developed in this study demonstrates superior discriminatory power compared to traditional clinical prediction tools. Indices such as the IPI, AAS, and ECOG performance status have long been used for risk stratification in DLBCL. However, these tools predominantly rely on a limited number of clinical variables, which inadequately capture the molecular heterogeneity among patients [7,33]. The findings of this study indicate that while clinical baseline models possess some efficacy in short-term risk prediction, their predictive capability diminishes over time. By incorporating transcriptomic features and using machine learning techniques, the model’s performance is significantly enhanced. This enhancement is evident not only in improved C-index and AUC values but also in the model’s refined ability to accurately identify high-risk groups, suggesting that the integrated model effectively addresses the limitations of traditional methods in heterogeneous diseases.

In the genomic analysis, we identified several candidate genes that are significantly associated with patients’ OS and recurrence risk. This study integrates 20 genes that showed statistical significance in univariate Cox analysis and further validates their prognostic value through stability selection and machine learning modeling. Notably, genes such as *PSMG4*, *CRY1*, *SPIRE1*, and *CKS1B* consistently appeared across multiple models, demonstrating uniform risk directionality, suggesting that they may be critical drivers in the prognosis of DLBCL. Unlike previous studies that focused solely on individual genes, this research emphasizes the stability and reproducibility of gene sets, enhancing the reliability of the results. Additionally, this study uncovered several potential molecular biomarkers that have been rarely reported before, providing new candidate targets for subsequent mechanistic research.

This study provides a clear comparison of various machine learning methods, highlighting the characteristics and applicability of different algorithms. The RSF model demonstrates certain advantages in short-term predictions, while GBSurvival exhibits balanced performance across multiple time points. FastSurvivalSVM stands out overall, aligning with findings from some hematologic malignancy prognosis studies that suggest support vector machines are advantageous in scenarios involving moderate-sized samples and high-dimensional features. This research further validates the rationale for algorithm selection, ensuring model robustness and generalizability through methods such as cross-validation and risk direction correction. These findings not only elucidate suitable contexts for different models but also offer significant technical references for future studies.

This study notably emphasizes the importance of model interpretability. Traditional machine learning models are often perceived as “black boxes,” which limits their acceptance in clinical practice [34,35]. By incorporating the SHAP method, this research clearly demonstrates the direction and magnitude of each feature’s contribution to model predictions. For instance, clinical indicators such as IPI score, AAS, and ECOG score continue to play a central role in the model, while molecular features like *PSMG4*, *CRY1*, and *LENEP* provide additional discriminative power. Through bar charts and beeswarm plots, we can intuitively understand how different feature values impact prediction outcomes, thereby enhancing the model’s transparency and clinical credibility. This methodological innovation ensures that machine learning predictions are not only accurate but also interpretable, which is crucial for advancing their clinical application.

In the realm of mortality risk prediction, the findings of this study align closely with existing literature. Traditional clinical factors such as age, LDH levels, ECOG performance status, and AAS remain significant in the multivariable Cox model, reaffirming their stability as fundamental prognostic indicators. However, by integrating molecular characteristics, the model’s efficacy in stratifying mortality risk is significantly enhanced, particularly in distinguishing between intermediate- and high-risk patients. This suggests that molecular abnormalities can complement clinical indicators, facilitate earlier identification of high-risk populations, and thereby provide a basis for intensified treatment or personalized follow-up strategies.

This study makes a significant contribution to the prediction of relapse risk. Previous research has predominantly focused on mortality outcomes, with insufficient systematic modeling of relapse or progression risk [36]. By integrating transcriptomic features, this study develops various relapse risk prediction models and demonstrates the superiority of FastSurvivalSVM. The analysis reveals that the drivers of relapse risk differ partly from those of mortality risk; for instance, genes such as *TMEM182*, *SPIRE1*, and *CKS1B* play a more prominent role in predicting relapse. This finding suggests that mortality and relapse in DLBCL may be driven by distinct molecular mechanisms, underscoring the necessity of dual prediction models. It also provides new insights for early intervention strategies targeting high-risk populations for relapse.

The findings of this study hold potential value for clinical translation. The model’s consistent performance in both short-term and long-term predictions suggests its use in aiding risk stratification and therapeutic decision-making at the initial diagnosis stage for patients. Particularly, the precise identification of high-risk patients can inform the selection of more aggressive treatment strategies, such as intensified immunotherapy or novel targeted drugs. Conversely, identifying low-risk patients can help avoid overtreatment and the associated toxic burden. Additionally, the visualization of risk scores facilitates clinicians in explaining prognostic outcomes to patients and their families during clinical consultations, thereby enhancing treatment adherence and the efficiency of follow-up management.

From a scientific research perspective, this study is closely linked to precision medicine and integrative multiomics research. In recent years, integrative analyses based on multiomics have increasingly become a crucial approach for elucidating complex disease mechanisms and achieving personalized management [37,38]. This study establishes a transferable framework for DLBCL risk prediction by integrating clinical data with transcriptomic features. This not only provides a tool for clinical risk assessment but also offers a set of candidate genes for exploring the molecular mechanisms of DLBCL. Future research could build upon this foundation by incorporating proteomics, immunomics, or spatial transcriptomics data to develop a more comprehensive and multidimensional risk prediction system.

In conjunction with the key genes identified by the model, this study also offers insights into potential biological mechanisms. For instance, *SMG4* (PAC4) is a proteasome 20S core particle assembly chaperone that participates in proteasome biogenesis and may therefore contribute to tumor-associated protein degradation by regulating proteostasis and stress adaptation [39]. *TMEM182* has been reported to be associated with tumor cell motility and migration in certain solid tumors, and accumulating evidence also suggests its involvement in differentiation programs and metabolic phenotype alterations [40,41]. These findings imply that *TMEM182* may contribute to the progression of DLBCL by modulating tumor cell adaptability or interactions with the tumor microenvironment.

Despite the systematic results obtained in prognostic modeling and interpretability analysis for DLBCL, several nonnegligible limitations of this study should be acknowledged. First, the analyses were conducted based on a single publicly available transcriptomic dataset (GSE31312). Although this cohort is relatively large, well-annotated with clinical information, and processed using a unified analytical pipeline, its single-source nature may still introduce potential selection bias and limit population heterogeneity. Consequently, the external generalizability of the proposed models may be constrained. The robustness and clinical applicability of these models across diverse populations and real-world settings, therefore, warrant further validation using independent external cohorts and multicenter prospective studies. Second, while multiple candidate genes significantly associated with overall survival and relapse risk were identified, their specific biological functions and molecular mechanisms in DLBCL pathogenesis and progression have not yet been experimentally validated. Future studies integrating Clustered Regularly Interspaced Short Palindromic Repeats-Cas9-mediated gene editing or RNA interference approaches in both in vitro and in vivo models could systematically evaluate the roles of these genes in tumor proliferation, invasion, and therapeutic response, thereby facilitating the translation of statistically associated biomarkers into potential functional targets. Finally, although rigorous modeling strategies, including cross-validation, robust feature selection, and multimodel comparisons, were used to mitigate overfitting, the predictive performance of machine learning models remains inherently dependent on the quality and dimensionality of the input data. Future work would benefit from incorporating additional multi-omics layers (such as somatic mutation profiles, copy number variations, and epigenetic features), as well as dynamic clinical variables related to treatment response and longitudinal follow-up. Such integration may enable the development of more comprehensive and dynamic risk stratification frameworks with improved predictive accuracy and clinical relevance.

In summary, this study systematically constructed and compared mortality and relapse risk prediction models for patients with DLBCL by integrating clinical features with transcriptomic data using various machine learning methods. The results demonstrated that FastSurvivalSVM exhibited superior overall performance and was capable of identifying key driving factors and clinical-molecular interactions through the SHAP method. Scientifically, this study proposes a robust multidimensional modeling framework, providing new evidence for understanding the biological heterogeneity of DLBCL. Clinically, it offers potential tools for individualized risk assessment, treatment decision-making, and follow-up management of patients. Despite limitations such as insufficient sample sources and external validation, this study lays the groundwork for future validation based on multicenter prospective data and the deep integration of multiomics data. With the continuous advancement of artificial intelligence and precision medicine, interpretable machine learning models are expected to play an increasingly important role in risk prediction and personalized management of lymphoma and other complex tumors.

## Appendix

Multimedia Appendix 1 Additional visual data.

Multimedia Appendix 2 Clinical characteristics of the included patients.

Multimedia Appendix 3 Overall survivial forest clinical multivariable results.

Multimedia Appendix 4 Overall survival single-gene Cox univariate results.

Multimedia Appendix 5 Relapse-free survival forest clinical multivariable results.

Multimedia Appendix 6 Final 20 genes.

Multimedia Appendix 7 Graphical Abstract. AI-driven prognostic risk prediction model for DLBCL integrating clinical variables and transporter transcriptomics to evaluate relapse and mortality risk.

## Abbreviations

AAS: Ann Arbor stage
AUC: area under the curve
DCA: decision-curve analysis
DLBCL: diffuse large B-cell lymphoma
ECOG: Eastern Cooperative Oncology Group
FDR: false discovery rate
FastSurvivalSVM: fast survival support vector machine
GBSurvival: gradient boosting survival
GEO: Gene Expression Omnibus
GEP: gene expression profiling
HR: hazard ratio
IPI: international prognostic index
LDH: lactate dehydrogenase
OS: overall survival
RFS: relapse-free survival
ROC: receiver operating characteristic
RSF: random survival forest
SHAP: Shapley Additive Explanations

## Data Availability

All data generated or analyzed during this study are included in this article and/or its supplementary material files. Further enquiries can be directed to the corresponding author.
