# Supplementary material for: Integrated Clinical-Molecular Risk Stratification in Diffuse Large B-Cell Lymphoma: Machine Learning Survival Analysis
Source: JMIR Med Inform. 2026 Aug 21;14:e84636. doi: 10.2196/84636 (PMC13545529; doi:10.2196/84636)
Supplement: Supplementary file 1 [file medinform_v14i1e84636_app1.docx]

**Figure S1. Overall analytical workflow of data integration and survival modeling in DLBCL.**

Note: The study is based on the GSE31312 dataset, integrating clinical characteristics and transcriptomic data. After data preprocessing, Cox regression analysis, gene selection, machine learning modeling, and interpretability analyses are sequentially performed, ultimately generating predictive results for mortality and relapse risk.

**Figure S2. Gene-level association analysis with OS.**

Note: (A) Schematic diagram of candidate gene selection and feature construction in DLBCL; (B) Forest plot of univariate Cox regression for 20 candidate genes, with several HRs deviating significantly from 1; (C) Kaplan–Meier survival curves show that patients with high expression of *PSMG4*, *CRY1*, *CRTAM*, *CTBP2*, *HHEX*, and *CASC9* have significantly worse OS.

**Figure S3. Supplementary calibration and decision curve validation of GBSurvival and RSF models for RFS.**

**Note: (A–B) Calibration curves of the GBSurvival model (A) and the RSF model (B) at 1, 3, and 5 years demonstrate good agreement between predicted probabilities and observed relapse rates; (C–D) DCA of the GBSurvival model (C) and the RSF model (D) show consistently higher net benefit compared with the treat-none and treat-all strategies across a range of threshold probabilities.**

**Figure S4. Supplementary risk stratification performance of GBSurvival and RSF models for RFS.**

**Note: (A–B) Risk score distributions and survival status plots of the GBSurvival model (A) and the RSF model (B) indicate that relapse events cluster predominantly in the high-risk group; (C–D) Kaplan–Meier curves stratified by median risk score for the GBSurvival model (C) and the RSF model (D) demonstrate significant separation of relapse risk between high- and low-risk groups; (E–F) Kaplan–Meier curves stratified by tertiles of risk score for the GBSurvival model (E) and the RSF model (F) further show highly significant stepwise differences among low-, intermediate-, and high-risk groups.**

**Figure S5. Gene-level association analysis with RFS.**

Note: (A) Univariate Cox regression forest plot of 20 candidate genes shows that *TMEM182*, *SPIRE1*, *CKS1B*, *MIR30D*, and *MDK* have HRs significantly deviating from 1; (B) Kaplan–Meier survival curves demonstrate that high expression of these genes is associated with markedly shorter RFS.

**Figure S6. Additional calibration and DCA analyses for GBSurvival and RSF models.**

Note: (A–B) Calibration curves of the GBSurvival model (A) and the RSF model (B) at 1, 3, and 5 years indicate good agreement between predicted probabilities and observed relapse rates; (C–D) DCA of the GBSurvival model (C) and the RSF model (D) show consistently higher net benefit than the treat-none and treat-all strategies across different threshold probabilities.

**Figure S7. Extended risk stratification analyses of GBSurvival and RSF models.**

Note: (A–B) Risk score distributions and survival status plots of the GBSurvival model (A) and the RSF model (B) indicate that relapse events cluster predominantly in the high-risk group; (C–D) Kaplan–Meier curves stratified by median risk score for the GBSurvival model (C) and the RSF model (D) demonstrate significant separation of relapse risk between high- and low-risk groups; (E–F) Kaplan–Meier curves stratified by tertiles of risk score for the GBSurvival model (E) and the RSF model (F) reveal highly significant stepwise differences among low-, intermediate-, and high-risk groups.
